# Psychiatric disorders prior to amyotrophic lateral sclerosis

**DOI:** 10.1002/ana.24801

**Published:** 2016-11-14

**Authors:** Martin R. Turner, Raph Goldacre, Kevin Talbot, Michael J. Goldacre

**Affiliations:** ^1^ Oxford University Nuffield Department of Clinical Neurosciences, John Radcliffe Hospital; ^2^ Oxford University Nuffield Department of Population HealthOld Road CampusOxfordUnited Kingdom.

## Abstract

It is recognized that neuropsychiatric conditions are overrepresented in amyotrophic lateral sclerosis (ALS) patient kindreds and psychiatric symptoms may precede the onset of motor symptoms. Using a hospital record linkage database, hospitalization with a diagnosis of schizophrenia, bipolar disorder, depression, or anxiety was significantly associated with a first diagnosis of ALS within the following year. This is likely to specifically reflect the clinicopathological overlap of ALS with frontotemporal dementia. A diagnosis of depression was significantly associated with a first record of ALS ≥5 years later, in keeping with growing evidence for major depressive disorder as an early marker of cerebral neurodegeneration. Ann Neurol 2016;80:935–938

The neurodegenerative disorder amyotrophic lateral sclerosis (ALS) is a syndrome involving clinical, histopathological, and genetic overlap with frontotemporal dementia (FTD).^1^, ^2^ This has brought increasing interest in the psychiatric manifestations of ALS, and there is a broader need to understand the wider at‐risk population if future neuroprotective strategies are to be usefully applied. We therefore sought to explore the prior occurrence of psychiatric diagnoses in relation to a subsequent diagnosis of ALS using an unbiased large hospital record linkage database.

## Patients and Methods

The method has been described in detail elsewhere.^3^ In essence, an all‐England national record‐linkage dataset of Hospital Episode Statistics and mortality data (1999–2011) was used to undertake studies of cohorts of people with several major psychiatric disorders, compared with a reference (control) cohort without these diseases, to determine the risk of subsequent ALS (International Classification of Diseases, Tenth Edition [ICD‐10] G12.2) in each cohort. The psychiatric disorders chosen were schizophrenia (narrowly defined as ICD‐10 F20.0–F20.9 and broadly defined as F20–F29), bipolar affective disorder (narrowly defined as ICD‐10 F31 and broadly defined as ICD‐10 F31 or F39), depression (ICD‐10 F32.0–F32.2 or F32.8–F32.9 or F33.0–F33.2), and anxiety (ICD‐10 F40–F41). In each analysis, the reference cohort comprised people without a known record of the relevant "exposure" diagnosis, and whose principal reason for admission to hospital was for any one of a wide range of conditions and operations comprising any of the following conditions/operations: cataract, otitis, varicose veins, hemorrhoids, upper respiratory tract infections, nasal polyp, deflected septum, tooth disorders, inguinal hernia, nail diseases, sebaceous cyst, internal derangement of knee, bunion, contraceptive management, selected limb fractures, selected dislocations, sprains and strains, gall bladder disease, dilation and curettage, appendectomy, hip replacement, and knee replacement. Anyone known to have a diagnosis of ALS on or before the record of psychiatric disorder or reference condition was excluded from the study. For each psychiatric cohort, the rate of ALS incidence was calculated based on person‐time from the earliest known relevant psychiatric record to the earliest known ALS record. Anyone who died was censored in the analysis. The rate of ALS in the reference cohort was calculated in the same way. In each analysis, rates were indirectly standardized to a population that comprised the psychiatric cohort and the reference cohort combined, stratified by age in 5‐year groups, sex, year of cohort entry, government office region of residence, and area‐level deprivation. This enabled calculation of (1) expected numbers of ALS patients in each cohort to compare with the numbers observed; and (2) standardized rate ratio estimates, based on the formula (O^psych^/E^psych^)/(O^ref^/E^ref^), where O and E are the observed and expected numbers of ALS in each psychiatric cohort and the reference cohort, respectively. Further analyses were conducted to determine whether the standardized rate ratios were modified by the interval of follow‐up after cohort entry. Analysis of the data was approved by the English National Health Service Central Office for Research Ethics Committees (reference number 04/Q2006/176).

## Results

Results are summarized in the Table. The numbers of people who entered the cohorts were as follows: schizophrenia (narrowly defined), 151,026; schizophrenia (broadly defined), 224,978; bipolar (narrowly defined), 85,799; bipolar (broadly defined), 89,469; depression, 843,820; anxiety, 356,602; reference cohort, 8,679,473. Hospitalizations with a first diagnosis of schizophrenia, bipolar disorder, depression, or anxiety were all significantly associated with diagnosis of ALS within the following year. Between 1 and 4 years before the first record of ALS, only bipolar disorder, depression, and anxiety were associated. A diagnosis of depression was significantly associated with a first record of ALS ≥5 years later.

**Table 1:** Observed and Expected Numbers of Patients in the Cohorts of People with Psychiatric Disorders (O^psych^, E^psych^) and in the Reference Cohort (O^ref^, E^ref^) Who Had a Subsequent Hospital Diagnosis of Amyotrophic Lateral Sclerosis (<1 Year Later, 1–4 Years Later, and 5 + Years Later)

Time Interval	Exposure Cohort	O^psych^	E^psych^	O^ref^	E^ref^	RR	95% CI	*p*
< 1 year	Schizophrenia (narrow)	26	9.9	691	707.1	2.69	1.74–3.97	<0.0001
	Schizophrenia (broad)	45	15.9	691	720.1	2.95	2.13–4.00	<0.0001
	Bipolar (narrow)	20	6.4	690	703.6	3.20	1.94–4.97	<0.0001
	Bipolar (broad)	23	6.7	689	705.3	3.54	2.23–5.35	<0.0001
	Depression	320	98.1	650	871.9	4.37	3.81–5.01	<0.0001
	Anxiety	121	37.8	681	764.2	3.59	2.93–4.36	<0.0001
1–4 years	Schizophrenia (narrow)	36	29.2	2,327	2,333.8	1.24	0.86–1.72	0.24
	Schizophrenia (broad)	56	44.0	2,323	2,335.0	1.28	0.96–1.67	0.08
	Bipolar (narrow)	34	18.0	2,323	2,339.0	1.91	1.32–2.67	0.0002
	Bipolar (broad)	37	18.6	2,322	2,340.4	2.01	1.41–2.78	<0.0001
	Depression	354	171.1	2,199	2,381.9	2.24	2.00–2.51	<0.0001
	Anxiety	131	71.7	2,288	2,347.3	1.87	1.56–2.24	<0.0001
5 + years	Schizophrenia (narrow)	24	23.2	1,796	1,796.8	1.03	0.66–1.54	0.96
	Schizophrenia (broad)	36	33.3	1,793	1,795.7	1.08	0.76–1.50	0.70
	Bipolar (narrow)	15	13.2	1,793	1,794.8	1.14	0.64–1.89	0.71
	Bipolar (broad)	15	13.5	1,792	1,793.5	1.11	0.62–1.84	0.79
	Depression	118	80.3	1,663	1,700.7	1.50	1.24–1.81	<0.0001
	Anxiety	43	31.8	1,761	1,772.2	1.36	0.98–1.84	0.05

Expected numbers are based on indirect standardization with the combined exposed and reference cohorts. The rate ratio is calculated from the formula (O/E) in the exposure cohort divided by (O/E) in the reference cohort.

CI = confidence interval; E = expected; O = observed; RR = standardized rate ratio.

## Discussion

Neuropsychiatric conditions may be overrepresented in ALS patient kindreds,^4^ and patient‐ and carer‐reported psychiatric symptoms have been shown to precede the onset of motor symptoms.^5^ Caveats generic to the less biased methodology used in this study are principally the reliance on accuracy of hospital coding, although expected to be high for a specialized disorder such as ALS, and also that the method only captures hospitalized (and not outpatient) events. An element of surveillance bias is possible: the possibility that ALS is diagnosed as a consequence of people with the psychiatric disorders being under medical supervision. However, any effect would be simply to make the diagnosis a little earlier.

The association of multiple major psychiatric conditions in the year prior to hospitalization with ALS is assumed to reflect psychotic symptoms associated with the clinicopathological overlap of ALS with FTD. Up to 15% of ALS patients in population‐based analysis fulfil criteria for dementia,^6^ and in those affected, this is typically an early symptom. At least 90% of cases of ALS appear to be sporadic. However, the commonest cause of both familial ALS and FTD is a hexanucleotide GGGGCC expansion in *C9orf72*, which may be associated with relatively pure forms of ALS and FTD, or their combination (ALS‐FTD) within the same pedigree.^7^ In the latter group, behavioral change is typically one of the first clinical features, and may include frank psychosis,^8^ such as might be misdiagnosed as schizophrenia or bipolar disorder. Whereas a limited association of this gene mutation has been reported with bipolar disorder^9^ and schizophrenia,^10^ the *C9orf72* expansion is estimated to be present in <10% of apparently sporadic ALS patients in Western populations.^11^ We therefore concur with the view that early psychiatric symptoms may reflect an inherent feature of ALS pathology beyond *C9orf72*‐related ALS‐FTD.^5^ More florid psychotic symptoms warrant further study, as they will impact on compliance with ALS‐related interventions such as gastrostomy and noninvasive ventilation, as well as on carer burden.^12^

The association of prior admission for schizophrenia, bipolar disorder, and anxiety with a subsequent diagnosis of ALS diminished with increasing distance from diagnosis, supports the view that the psychiatric symptoms are likely to be part of the motor prodromal landscape, rather than a long‐term trait. Depression, however, remained a significant remote psychiatric comorbidity ≥5 years prior to the onset of ALS. This latter association has been recognized in Parkinson disease^13^ and dementia more broadly.^14^, ^15^ The presymptomatic landscape in ALS, through the study of carriers of high‐penetrance mutations, has become a focus of interest to understand the earliest pathogenic changes, and begin the move toward primary prevention.^16^ Structural^17^ and functional^18^ cerebral changes have been demonstrated in asymptomatic ALS gene carriers. There is increasing evidence linking pathways involved in central nervous system development, such as extensive cerebral neuronal pruning in early adult life, with late life neurodegeneration.^19^ Neuroimaging studies of brain structural networks indicate a shared vulnerability between schizophrenia and Alzheimer disease.^20^ Evidence supporting shared neurodevelopmental risk factors between ALS and complex neuropsychiatric disorders of early life has arisen from a surrogate marker of intrauterine testosterone exposure.^21^ The present study raises the possibility that the development of depression in middle age may be a specific risk factor for neurodegenerative disorders. It is one that could be screened for in primary care to provide a subpopulation on which to focus future neuroprotective strategies.^22^

## Author Contributions

All authors contributed to study concept and design, to data acquisition and analysis, and to drafting the manuscript.

## Potential Conflicts of Interest

Nothing to report.
